# Highlight selection of radiochemistry and radiopharmacy developments by editorial board

**DOI:** 10.1186/s41181-025-00335-w

**Published:** 2025-03-25

**Authors:** S. Spreckelmeyer, J. Dasilva, C. Decristoforo, R. H. Mach, J. Passchier, G. Carlucci, M. Al Qahtani, A. Duatti, B. T. Cornelissen, J. Engle, A. Denkova, J. J. M. A. Hendrikx, Y. Seimbille, X. Yang, H. Jia, M-R. Zhang, M. Yang, L. Perk, P. Caravan, P. Laverman, Z. Cheng, C. Hoehr, T. Sakr, J. R. Zeevaart

**Affiliations:** 1 Department of Nuclear Medicine, Charité - Universitätsmedizin Berlin, Corporate Member of Freie Universität Berlin, Humboldt- Universität zu Berlin, Berlin Institute of Health, Augustenburger Platz 1, 13353 Berlin, Germany; 2 University of Montreal, Montreal, Canada; 3 Medical University Innsbruck, Innsbruck, Austria; 4 University of Pennsylvania, Philadelphia, USA; 5 Invicro London, London, UK; 6 University of California, Los Angeles, CA USA; 7 Cyclotron and Radiopharmaceuticals Department, Research & Innovation, King Faisal Specialist Hospital and Research Center, Riyadh, Saudi Arabia; 8 University of Ferrara, Ferrara, Italy; 9 Oxford University, Oxford, UK; 10 University Medical Center Groningen, Groningen, Netherlands; 11 University of Wisconsin, Madison, WI USA; 12 Delft University of Technology, Delft, Netherlands; 13 NKI, Amsterdam, The Netherlands; 14 Erasmus MC, Rotterdam, Netherlands; 15 Peking University First Hospital, Beijing, China; 16 Beijing Normal University, Beijing, China; 17 National Institutes for Quantum Science and Technology, Chiba, Japan; 18 Jiangsu Institute of Nuclear Medicine, Jiangsu, China; 19 Radboud University Medical Center Nijmegen, Nijmegen, Netherlands; 20 Massuchusetts General Hospital, Harvard University, Boston, USA; 21 Radboud Medical Center, Nijmegen, Netherlands; 22 Shanghai Institute of Materia Medica, Chinese Academy of Sciences, Shanghai, China; 23 TRIUMF, Vancouver, BC Canada; 24 Egyptian Atomic Energy Authority, Cairo, Egypt; 25 Necsa, Pretoria, South Africa

**Keywords:** Highlight articles, Radiochemistry, Radiopharmacy, Radiopharmaceutical sciences, Nuclear medicine

## Abstract

**Background:**

The Editorial Board of EJNMMI Radiopharmacy and Chemistry releases a biannual highlight commentary to update the readership on trends in the field of radiopharmaceutical development and application of radiopharmaceuticals.

**Main body:**

This selection of highlights provides commentary on 24 different topics selected by each co-authoring Editorial Board member addressing a variety of aspects ranging from novel radiochemistry to first-in-human application of novel radiopharmaceuticals.

**Conclusion:**

Trends in radiochemistry and radiopharmacy are highlighted. Hot topics cover the entire scope of EJNMMI Radiopharmacy and Chemistry, demonstrating the progress in the research field in many aspects.

## Background

Each individual co-authoring member of the Editorial Board has selected to highlight an article that has appeared in the radiochemistry, radiopharmacy and imaging agent literature during the period July-December 2024. The aim of this collaborative initiative is to create a biyearly overview for the readers summarizing the latest trends and hot topics in the field.

## Main text

### The effect of radiochemical precursors should not be neglected

By Sarah Spreckelmeyer

The study *“The importance of radiochemical purity: Cellular binding and internalization of different radiometal chlorides in prostate cancer cells”* (Raitanen et al. 2024) addresses the critical role of radiochemical purity in the evaluation of preclinical experiments. Focusing on prostate cancer cells (PC-3 and LNCaP), the authors examine how different radiometal chlorides behave in terms of cellular binding and internalization.

The methods used for [^111^In]InCl_3_, [^68^Ga]GaCl_3_ and [^177^Lu]LuCl_3_ are total cell binding, cell internalization assay, nuclei isolation assay and stability evaluation (only for [^177^Lu]LuCl_3_). Results showed that especially for [^177^Lu]LuCl_3_, the cell binding makes up to 25%. For [^111^In]InCl_3_ and [^68^Ga]GaCl_3_ they found, that up to 3% are internalized in the tested cell lines. The results demonstrate the importance of high radiochemical purity. Even if the radiopharmaceuticals or novel tracers under evaluation comply with common release-criteria, the radiochemical precursors– if present as impurities in the final product– can have a significant impact on the uptake results. The results emphasize the need for monitoring the effect of radiochemical precursors on the tested cell lines and to include them as controls in the respective preclinical experiments.

### A hydrophilic bifunctional Silicon-Based fluoride acceptor (SiFA)-based approach for producing radiohybrid radioligands

By Jean N. DaSilva

A radiohybrid approach that combines a chelator for radiometals (^68^Ga for diagnostic or ^177^Lu for β^–^ therapy) and a Silicon-Based Fluoride Acceptor (SiFA) moiety for ^18^F-PET imaging within the same molecule was successfully applied to produce peptide-based radiopharmaceuticals targeting the prostate-specific membrane antigen (PSMA) (Wurzer et al. 2020) or the cholecystokinin-2 receptor (Holzleitner et al. 2022). Deiser and colleagues recently reported a new hydrophilic bifunctional (SiFA)SeFe unit which enables straightforward and versatile linkage to biomolecules as it can be inserted both terminally and bridged via amide bond formation into radiohybrid ligands (Deiser et al. 2024). High stability to defluorination of the new (SiFA)SeFe moiety was observed under different conditions when incorporated in model bioconjugate peptides.

As proof-of-concept, three somatostatin receptor 2 (sstR2)-targeted radiohybrid constructs were prepared by incorporating the (SiFA)SeFe moiety either in a terminal ((SiFA)SeFerhTATE1 and (SiFA)SeFe-rhTATE3) or bridged ((SiFA)SeFe-rhTATE2) position. Theranostic compound (SiFA)SeFe-rhTATE3 could be labelled either with ^18^F for PET imaging or chelation with ^177^Lu for radiotherapy. All these compounds displayed high stability in human serum, high binding affinity toward sstR2 that was similar to that of [^68^Ga|Ga-DOTA-TATE, excretion *via* the kidneys and high accumulation in tumours with low bone uptake (i.e. low ^18^F^−^ defluorination) in ex vivo biodistribution studies in tumour-bearing CD1-nu/nu mice. Such properties provide appropriate basis to further evaluate such a versatile (SiFA)SeFe labelling approach for radiohybrid ligands.

### Improvements of PSMA radioligand therapy by using Tb-161 in combination with a new PSMA ligand– first in human data

By Clemens Decristoforo

PSMA-radioligand therapy has become an established therapeutic approach in patients with metastatic castration resistant prostate cancer (mCRPR), boosting the recognition of nuclear treatment strategies in the field of oncological therapies. To overcome current shortcomings, radiopharmaceutical research aims to increase the delivery of radiation dose and to achieve superior efficacy towards micro-metastases. One strategy is based on the use of Tb-161 because its high emission of both short-range conversion electrons and Auger electrons lead to increased radiation dose to micro metastases compared to Lu-177. The group of Christina Müller at PSI in Switzerland have developed a novel ligand, [¹⁶¹Tb]Tb-SibuDAB, which includes an albumin binder for prolonged circulation, resulting in enhanced tumour accumulation and radiation dose (Tschan et al. 2023). In a recent “Image of the Month” in EJNMMI, the group now reports on the first patient included in an elegantly designed prospective Phase 1a clinical trial undertaking the head-to-head comparison of the new [¹⁶¹Tb]Tb-SibuDAB with standard [^177^Lu]Lu-PSMA I&T (Chirindel et al. 2024). Quantitative SPECT/CT imaging was performed, and both the first dosimetry calculations and a comparison of pharmacokinetics were reported. As predicted, [¹⁶¹Tb]Tb-SibuDAB showed prolonged circulation and more than doubled radiation dose and tumour retention half-lives with favourable tumour/organ ratios for [¹⁶¹Tb]Tb-SibuDAB underlining its potential for radioligand therapy. This paper provides the first data in humans of a PSMA-targeted albumin binder in combination with Tb-161. More importantly, it underlines the importance of well-designed prospective clinical trials during the translation of novel developments in order to generate scientifically valid data even in a small number of patients. Many more clinical data are required before final conclusions on the clinically added value of [¹⁶¹Tb]Tb-SibuDAB in particular, and Tb-161 in general, can be made. However, these initial results are very promising.

### Room temperature photochemical Copper-Mediated fluorination of Aryl Iodides

By Bob Mach

Radiofluorination reactions referred to as “late-stage fluorination” have made a significant contribution to radiotracer synthesis in recent years. However, these reactions typically require high temperatures and specialized leaving groups on the precursor, such as boronates and stannanes, which can be difficult to synthesize, frequently offer poor chemical stability, and present toxicity challenges. Precursors containing an iodide leaving group have many advantages in ease of synthesis and chemical stability, but their use in radiofluorination has been limited by the need for harsh reaction conditions and low radiochemical yields. A recent paper has the potential to provide a novel approach for radiofluorination by utilizing a photochemical copper-mediated (radio)fluorination of aryl iodides (Spiller et al. 2024). This method, which involves irradiating the starting material with UV light at a λ_max_ of 313 nm, converts aryl iodides to aryl fluorides using fluorine-19 in yields up to 98%. The reaction uses copper and silver salts as mediators and is proposed to photolytically form an aryl radical (Ar·) followed by reaction with Cu(II)-F to yield the corresponding aryl fluoride. When applied to radiofluorination with no-carrier added fluorine-18, conversions were initially low (~ 4%). Addition of carrier fluorine-19 improved efficiency but was impractical because of the low molar activity of the product. To overcome this limitation, the authors found that substituting [^19^F]KF carrier with other anions (carbonate and phosphate salts) increased the radiochemical yield to 23–66% using a range of aryl iodide precursors. An important feature of this method is that the reaction proceeds at room temperature, which is typically not possible with radiofluorination. Expansion of this method to structurally diverse molecules is a key requirement for use in PET radiotracer development. Regardless, the mild reaction conditions and readily obtainable aryl iodide precursors demonstrate the potential utility of this method in PET radiochemistry.

### The advent of fibrosis imaging

By Jan Passchier

Significant efforts are underway to develop therapies against inflammation induced fibrotic disease such as lung, liver and kidney fibrosis. PET imaging has recently been made accessible by the development of tracers for targets such as fibroblast activation protein (FAP), collagen-1 (COL-1), platelet derived growth factor receptor beta (PDGRFβ) and mitochondrial complex-1 (MC1). Of these, tracers against FAP are in the most advanced stage of characterisation as evidenced by the highlighted paper (Bahtouee et al. 2025). In their paper, the authors report a significant difference in interstitial lung disease based on standard uptake value (SUV) measurement between patients and healthy controls using [^68^Ga]Ga-FAPI-46. These results are highly encouraging and offer future opportunities for future diagnostic use as well as understanding efficacy of novel treatments.

While this finding is promising, the SUV methodology applied points to a wider problem observed in the field. Clearly, the SUV approach works well for the application of [^18^F]FDG in oncology, especially if appropriately controlled for blood glucose levels. However, it is often overlooked that SUV as a simplified and quantitative measure for cancer imaging has been validated over the course of many decades of research. This cannot be assumed for novel tracers and care should be applied to demonstrate SUV measures will work equally well for new radiotracers. This requires full tracer characterisation using dynamic imaging, and an arterial or venous input function, to enable biomathematical modelling as the first step in understanding the true value of new imaging tools before exploring and validating simplified approaches.

### Preclinical evaluation of ^226^Ac as a theranostic agent: imaging, dosimetry, and therapy

By Giuseppe Carlucci

A new study evaluates the potential of ^226^Ac (t_1/2_ = 29.37 h) as a theranostic isotope, combining diagnostic imaging (158 and 230 KeV photons) with targeted therapy (4 high-energy particles) (Koniar et al. 2024). ^226^Ac was produced at TRIUMF by a 480 MeV proton beam bombarding a uranium carbide target. Following isolation from other spallation products, the isotope was labelled with the bioconjugate crown-TATE and assessed in AR42J tumour xenografts. Quantitative SPECT imaging revealed high tumour uptake and retention, with biodistribution studies indicating significant absorbed doses. Treatment with varying doses of ^226^Ac-crown-TATE significantly extended median survival, from 7 days in controls to up to 27 days in treated groups, without remarkable toxicities. Ex vivo biodistribution data were used to estimate the preclinical radiation dosimetry for the tumour and various organs of interest in the animal model. The tumour demonstrated the highest absorbed dose coefficient (222.3 mGy/ kBq), and all other reported organs were less than 80 mGy/kBq. The highest dose coefficients for normal organs were the stomach, kidneys, and pancreas, which warrants future studies investigating the maximum tolerated dose. Moreover, although the antitumor capabilities of ^226^Ac were shown, a complete response was not reached in any treated animal. This was likely due to low injected activities needing further optimization once off-target toxicities allow a higher injected dose.

This proof-of-concept study highlights ^226^Ac’s dual role as a diagnostic and therapeutic isotope, paving the way for future investigations into its clinical applications and optimizing treatment protocols for cancer patients.

### RESCA as powerful conjugated chelator for labelling affibody molecules with Fluorine-18

By Mohammed Al-Qahtani

The article discusses a new labelling methodology for Affibody molecules using [^18^F]AlF, a commonly used intermediate labelling agent for Positron Emission Tomography (PET). The research employs chelating chemistry and utilizes an efficient Restrained Complexing Agent (RESCA) as the chelator. The study demonstrates that chelator-based radiolabelling reactions can be conducted under mild conditions, leading to a radiolabelling process that is significantly simpler than many traditional radio-fluorination methods.

In this study, the researchers optimized the fluorine-18 radiolabelling of Affibody molecules using RESCA as an effective chelator, producing conjugated Affibody molecules with [^18^F]AlF. The article details the optimization process, covering various reaction temperatures and times, resulting in promising outcomes that underscore the advantages of this chelator. It is important to note that Cleeren F and his team initiated a generic radiolabelling method, highlighting its potential as a kit-based strategy for fluorine-18 labelling. This approach paves the way for developing numerous new fluorine-18-labelled protein-based radiotracers (Cleeren et al. 2017, 2018).The straightforward fluorine-18 labelling process has been fully optimized and can be widely applied to RESCA-conjugated Affibody molecules and similar peptide-based imaging agents.

**Fig. 1:**
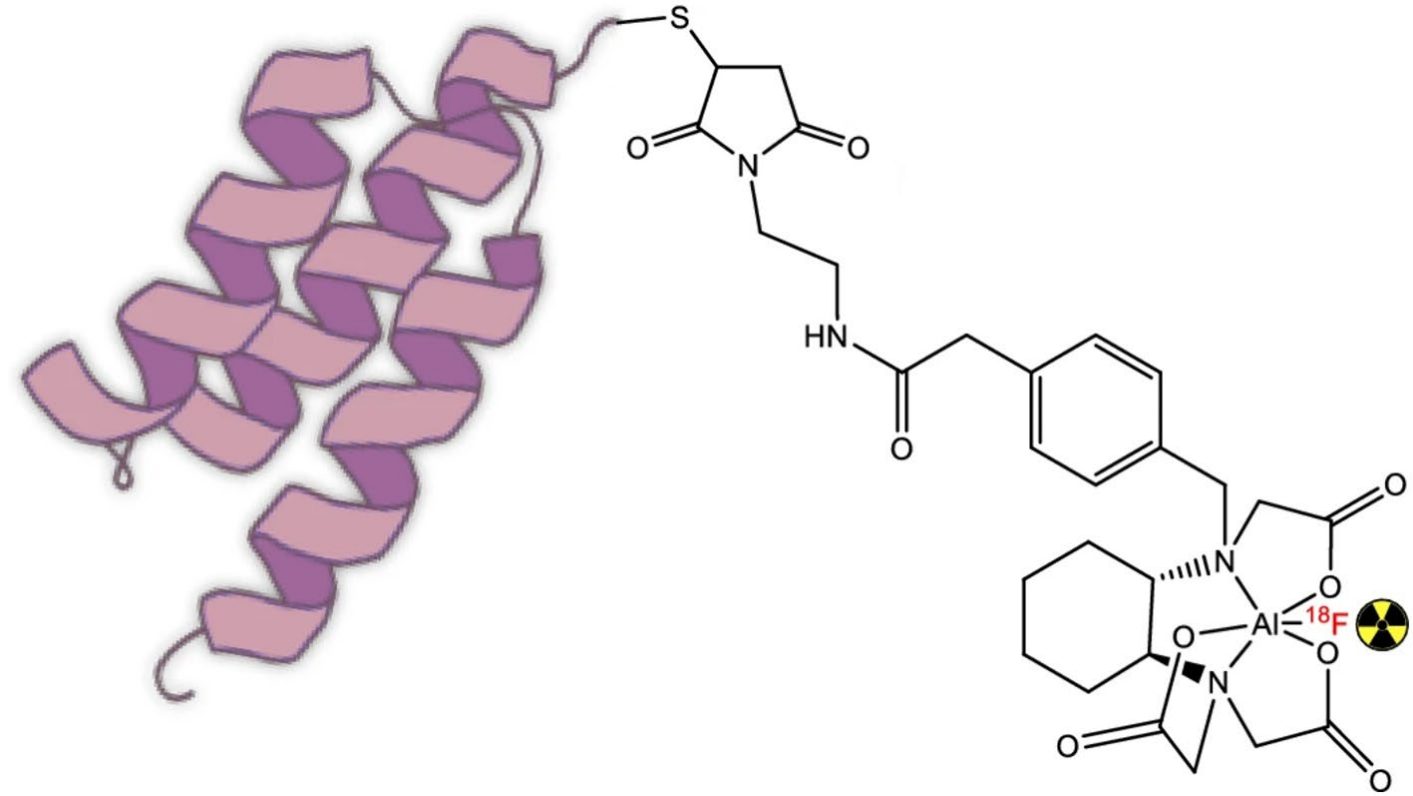
General structure of Al[^18^F]F labelled RESCA-conjugated Affibody molecules. Reprinted from Leschi et al. under a Creative Commons Attribution 4.0 International License (Lechi et al., 2024)

Two model Affibody proteins, Z09591 and Z0185, targeting PDGFRβ and TNFα, were chosen for this study and tested under optimal reaction conditions. Notably, the labelled compounds maintained their biological function, successfully targeting their corresponding molecules. Overall, the methodology is highly reproducible, efficient, and reliable. Figure 1 illustrates the general structure of Al^18^F-labelled RESCA-conjugated Affibody molecules.

### The molecular ‘pathology’ of theranostic radiopharmaceuticals

By Adriano Duatti

The dogmatic use of a purely mechanistic (Newtonian) representation of biomolecular interactions is becoming more of a pathological burden for the design of novel theranostic radiopharmaceuticals, rather than a useful methodological tool. The relentless search for new receptor targets and of drugs capable of binding to them selectively and specifically, is still based on the classical Anfinsen’s structure-function paradigm that a protein’s function is inextricably encrypted in strings of amino acids that fold into three dimensional pockets, helices and pleated sheets. However, a significant fraction of the proteome is populated by a polymorphic ensemble of intrinsically disordered proteins (IDPs) that are still capable of performing biological functions (Newcombe et al. 2024). Furthermore, the genome turned out to be no blueprint for an organism since most genes do not have a pre-set function that can be determined from their DNA sequence (Noble and Noble 2023). These findings show that AlphaFold was probably too late and raise questions about the universal validity of a naive molecular approach for designing new theranostic radiopharmaceuticals. In fact, it is discomforting that an effective and long-lasting radionuclide therapy against various cancers is still missing. Yet, nature occasionally points out a simple path to follow. The chemically simple positron and Auger emitter [^64^Cu]CuCl_2,_ has been found to be a useful theranostic agent for urinary tract neoplasms (Piccardo et al. 2024). The biological properties of [^64^Cu]CuCl_2,_ are mostly dictated by the natural physiological role of copper ions and may stimulate a switch from a crude molecular paradigm to a physiologically based approach.

### p53 stabilisation potentiates [^177^Lu]Lu-DOTATATE treatment in neuroblastoma xenografts

By Bart Cornelissen

Many stratagems are possible to boost the efficacy of targeted radionuclide therapy. This includes methods to increase target epitope expression, auto-amplification, or other means to boost radionuclide uptake in the target, tumour tissue, given that ‘more’ is ‘better.’ This of course is true, yet a less trodden path is the smart targeting of radiobiology to improve therapeutic efficacy.

Most tumours are tumours because of defects in oncogenes or tumour suppressor genes, resulting in an uncontrolled cell cycle, causing cancer cells to duplicate unchecked. The lynchpin in this context is TP53, the gene coding for the p53 protein, dubbed the ‘master regulator’ gene of a plethora of cellular processes. When the protein is mutated or muted, this control is lost. Indeed, nearly all tumours have disrupted p53 signalling, and a legion of strategies has been devised with p53 as a therapeutic target. In fact, targeted radionuclide therapy causes DNA damage and cell cycle effects, and we know that p53 is involved in cellular response to DNA damage and regulating the cell cycle.

The ability of the VIP116 peptide to inhibit the dampening effects of MDM2 and MDM4 on p53, by preventing them binding each other, to change its ability to direct the cells’ response to ^177^Lu-DOTATATE therapy was investigated (Berglund et al. 2024). This combination resulted in significantly better therapeutic efficacy, while not affecting normal tissue toxicity. VIP116 acts on intact, unmutated p53, which is the case for neuroendocrine tumours, a great match.

### Continued progress towards a challenging, longstanding objective: the incorporation chemistry of antimony radionuclides for targeted therapy

By Jonathan Engle

Radiochemical incorporation of no-carrier-added antimony into targeted drugs has been conspicuously elusive since a seminal in silico relation of low-energy electron emission spectra to cellular dosimetry has been published (Bernhardt et al. 2001). Despite the intriguing match identified between the range of ^119^Sb’s primary electronic emissions and typical cellular geometries, the chemistry of the element is relatively poorly understood, and this knowledge gap impedes exploration of an ^119^Sb-radiolabelled pharmaceutical. In over two decades, as the authors of the present study (Gé et al. 2024) remind poignantly, “*no suitable biocompatible chelating ligand system has been developed for delivering antimony*.” Noting reports of Sb(III) complexes with a tristhione scorpionate ligand hydrotris(methimazolyl)borate (TMe), a creative labelling strategy has been devised that straddles the indocile + 3 and + 5 oxidation states of the element and investigated the resulting complex’s stability, which persists up to ~ pH 4 (Gé et al. 2024). This is strong evidence that ongoing work can approach a fully bifunctional chelating paradigm that will enable us to experimentally convolve intercellular distribution with electron energy spectrum and observe effects on dose delivery and tumour microenvironment. The ultimate objective, understanding whether and how Auger and conversion electron-emitting radionuclides may one day fit into the rapidly expanding world of theranostic technologies, seems closer than it has yet.

### Pharmacokinetic modelling helps differentiating malignant and benign tissues when using FAPI tracers

By Antonia Denkova

Radiotracers based upon fibroblast activation protein inhibitors (FAPI) have shown to be excellent tracers for diagnosing cancer and are applicable to various cancer types (Mori et al. 2024). FAPIs are typically paired with positron-emitting radioisotopes to enable PET imaging. A key advantage of FAPI tracers are their ability to target cancer-associated fibroblasts, making them effective across a wide range of tumours rather than being restricted to a single type. However, FAPI uptake is not exclusive to malignant tissues, as it can also accumulate at inflammation sites (Boschi et al. 2024). While that can be considered an advantage, it also makes distinguishing between benign and malignant tumours rather difficult, particularly if only SUV (standardized uptake value) is used for identification.

To address these challenges, Chen et al. demonstrated that pharmacokinetic modelling based on total-body PET imaging scans could enhance the accuracy of cancer diagnosis (Chen et al. 2024). Their recent study involved 113 patients using [^68^Ga]Ga-FAPI-04, and it revealed that three dynamic modelling parameters—biomarkers for blood volume in tumours, perfusion, and receptor binding combined with internalization—can effectively differentiate between malignant and benign tissues. Among these, the blood volume parameter was the most predictive, consistent with the expectation that malignant tumours are typically better vascularized and exhibit higher fractional blood volume.

This study highlights the significant potential of pharmacokinetic modelling in improving diagnostic precision. By combining total-body PET imaging and dynamic modelling, it becomes possible to more reliably identify malignant tissues. The authors also emphasize that improving imaging protocols can further enhance pharmacokinetic modelling, making this approach even more attractive for future clinical applications.

### Comparison of the tolerability of ^161^Tb- and ^177^Lu-labelled somatostatin analogues in the preclinical setting

By Jeroen Hendrikx

The interest in the radionuclide terbium-161 (^161^Tb) as a substitute for lutetium-177 (^177^Lu) is rising. ^161^Tb has similar decay properties as ^177^Lu, although co-emission of conversion and Auger electrons may favour use of ^161^Tb over ^177^Lu (Müller et al. 2023). A switch in radionuclide may improve the treatment efficacy of somatostatin analogues used for targeted radionuclide therapy. In vitro data indeed show higher dose response rates of ^161^Tb (De Nardo et al. 2024), resulting in improved preclinical efficacy of ^161^Tb-labelled analogues (Borgna et al. 2022). However, this may come with decreased tolerability of the radionuclide therapy. Busslinger and colleagues evaluated preclinical tolerability of [^161^Tb]Tb-DOTATATE and [^161^Tb]Tb-DOTA-LM3 and the ^177^Lu-labelled counterparts in mice (Busslinger et al. 2024). A main focus was on haematological toxicity, although histopathological evaluation of other organs was also performed. The results showed good tolerability of a low dose of the ^161^Tb-based analogues (20 MBq, comparable to a human dose of ~ 4.5 GBq) and even direct comparison of ^161^Tb-labelled analogues (100 MBq) with ^177^Lu-labelled analogues (100 MBq) showed comparable tolerability. This indicates that clinically administered activity of ^161^Tb may be increased to similar levels as currently used for ^177^Lu and thus may increase efficacy as it leads to higher absorbed tumour doses. The authors justly note that transferability to the human situation is not clear, however, these results justify proper dose-escalation studies with ^161^Tb-labelled analogues to study tolerability and dosimetry in patients to optimize future treatment efficacy, rather than simply dose selection based on absorbed tumour dose of ^177^Lu-labelled analogues.

### Can pretargeting achieve the optimal balance between efficacy and off-target toxicity?

By Yann Seimbille

Single-domain antibodies (sdAbs) have favorable pharmacokinetic properties for molecular imaging, but their significant kidney accumulation poses a major obstacle to their application in targeted radionuclide therapy. To address this limitation, Poty et al. implemented a click-chemistry–based pretargeting strategy to reduce kidney uptake of the fibroblast activation protein (FAP)-targeted sdAb, 4AH29 (Poty et al. 2024). Key parameters were optimized in FAP-positive U87MG tumor-bearing mice, including the injected mass of 4AH29-TCO (200 µg) and the lag time (8 h) between the injections of 4AH29-TCO and [^177^Lu]Lu-DOTA-PEG_7_-tetrazine. It led to a five-fold higher tumor-to-kidney ratio than that of the radiolabeled sdAb alone (2.4 vs. 0.45 for [^177^Lu]Lu-DOTA-4AH29). The pretargeting strategy was subsequently tested in a pancreatic ductal adenocarcinoma patient-derived xenograft model, which better mimics tumor heterogeneity and stromal structure compared to U87MG xenografts, but exhibits significantly lower FAP expression. Despite the low tumor mean absorbed dose due to the poor FAP expression, pretargeting delayed tumor growth and extended survival in the pretargeted cohort (3 × 88 MBq) compared to controls. Importantly, no kidney morphologic changes were observed with pretargeting, whereas the conventional approach led to mesangial expansion. This study highlights the potential of pretargeting to reduce off-target toxicity and preserve healthy tissues. However, it also underscores the need for animal models that more accurately reflect clinical conditions.

### Targeted positron emission tomography-tracked biomimetic codelivery synergistically amplifies ferroptosis and pyroptosis for inducing lung cancer regression and anti-PD-L1 immunotherapy efficacy

By Xing Yang

The chemoresistance and systemic toxicity of cisplatin (CDDP) severely limit its application in the treatment of non-small cell lung cancer (NSCLC) (Valdes et al. 2016). Recently, a research team from Peking University conducted a study on ^124^I-labelled cancer cell membrane biomimetic nanovesicles loaded with Polyphyllin VI (PPVI) and CDDP (termed I–P/C@CMLvs) (Zhu et al. 2024). The authors developed a highly innovative approach that tackles NSCLC through the synergistic amplification of ferroptosis and pyroptosis, two forms of regulated cell death pathways, thereby amplifying the cytotoxic effects on lung cancer cells. The results showed that the I–P/C@CMLvs promoted deubiquitylation of p53 and stimulated reactive oxygen species generation, which triggers a crosstalk between GPX4 signalling pathway and NLRP3/GSDMD/Caspase-1 axis. This study, which incorporated targeted PET tracking, not only demonstrates the distribution and drug delivery of nanoparticle to ensure targeted treatment and minimize systemic toxicity, but also provides a framework for evaluating therapeutic efficacy in real time.

Besides, by promoting immunogenic cell death (ICD), the combination of I–P/C@CMLvs and anti-PD-L1 therapy further promotes NSCLC regression, which may prevent tumour recurrence. Therefore, it is a potentially strategy that could combine therapies integrating advanced imaging, targeted drug delivery, and immunotherapy for various cancers, with promising clinical translation prospects.

### Unleashing the power of radiopharmaceuticals for prodrug activation

By Hongmei Jia

Chemotherapy remains a powerful tool in combating cancer, but its effectiveness is often hampered by systemic toxicity. Tumour-selective prodrug activation offers a promising approach to alleviating this challenge (Fu et al. 2023). Radiopharmaceuticals, labelled with radionuclides, hold increasing promise for diagnosis and treatment of cancer metastases. However, the concept of radiopharmaceutical-induced prodrug activation has not yet been established.

**Fig. 2:**
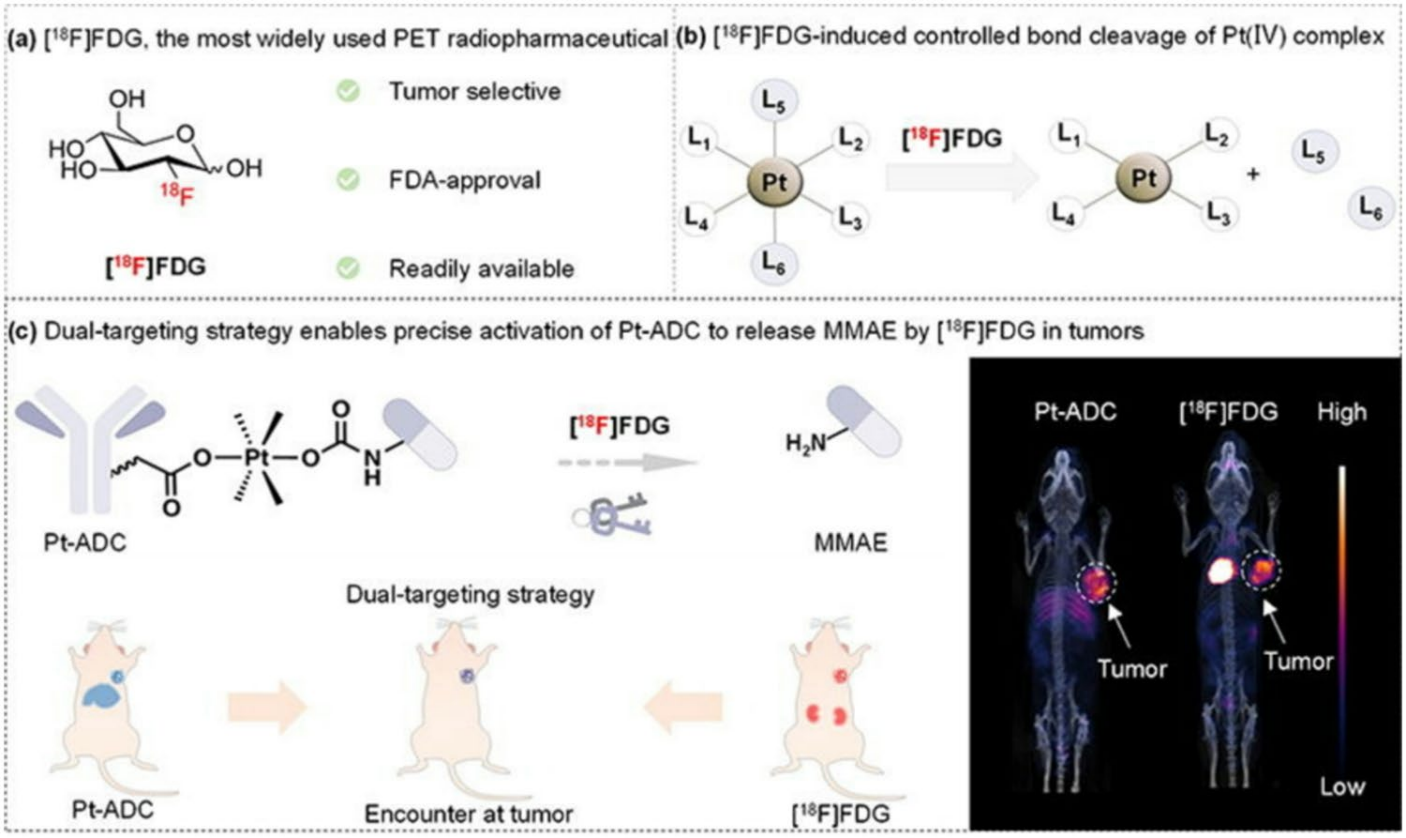
[^18^F]FDG unlocks Pt(IV) prodrugs in tumours. (**a**) [^18^F]FDG is a tumour-selective and prevalent PET tracer. [^18^F]FDG accumulates in tumours selectively due to the Warburg effect, exhibiting good pan-cancer diagnostic performance in clinics. (**b**) [^18^F]FDG triggers Pt(IV) complex to readily release biological function axial ligands and Pt(II) drugs. (**c**) The dual-targeting strategy enables precise activation of Pt-ADC by [^18^F]FDG at tumours. Reproduction permitted via Rightslink from Wang C, Xu M, Zhang Z, Zeng S, Shen S, Ding Z, Chen J, Cui XY, Liu Z. Locally unlocks prodrugs by radiopharmaceutical in tumour for cancer therapy. Sci Bull. 2024;69:2745–2755. ^©^ Elsevier

Recently, researchers from Peking University (Wang et al. 2024a, b) revealed an innovative strategy of improved tumour-specific prodrug activation by a tumour-selective radiopharmaceutical (Fig. 2). In this study the authors selected [^18^F]FDG, the most widely used PET radiopharmaceutical, to serve as a generally applicable tool to trigger the Pt(IV) complexes to release the axial ligands and Pt(II) drugs, possibly mediated by the hydrated electrons generated via water radiolysis during the decay of fluorine-18. This controlled release reaction occurs efficiently in both test tubes and living cells. Based on these findings, an OxaliPt(IV)-based linker has been developed to create an [^18^F]FDG-activated antibody-drug conjugate (Pt-ADC). PET/CT imaging results demonstrate colocalization of [^18^F]FDG and Pt-ADC in the tumour, enabling tumour-specific prodrug activation through this dual-targeting strategy. [^18^F]FDG precisely unlocks Pt-ADC and thus effectively suppresses tumour growth in tumour-bearing mice without significant side effects and offers a promising strategy for treating cancer metastases. Finally, it is also interesting that [^18^F]FDG induces the deprotection of various radiotherapy removable protecting groups (RPGs). In a word, unleashing the power of radiopharmaceuticals for prodrug activation in primary tumour and metastasis (Guo et al. 2024) may pave new ways for developing innovative cancer management strategies.

### D-[^18^F]fluoroalanine and D‐[^18^F]fluoroalanine‐*d*_*3*_ for PET imaging of bacterial infection

By Ming-Rong Zhang.

D-alanine is a substrate for biosynthesis of bacterial peptidoglycan and can be selectively taken up by bacteria and not mammalian cells. D-amino acid metabolism is thus an attractive target for the development of bacteria-specific PET imaging agents and discovery of new antibiotics. D-3-[^18^F]Fluoroalanine (D-[^18^F]FAla) and D-3-[^18^F]fluoroalanine-*d*_*3*_ (D-[^18^F]FAla-*d*_*3*_) were synthesized using stereo-specific cyclic sulfamidates as two radiolabelling precursors, and evaluated their potential to visualize bacterial infection (Li et al. 2024). In vitro bacterial uptake study showed that D-[^18^F]FAla and D-[^18^F]FAla-*d*_*3*_ were taken up specifically by the Gram-negative *E. coli* and Gram-positive *S. aureus*. A biodistribution study on a soft-tissue model of *S. aureus* infection revealed that the radioactive uptakes of D-[^18^F]FAla and D-[^18^F]FAla-*d*_*3*_ reached 0.64 − 0.78% ID/cc in the infectious muscles at 15 min post-injection, which were significantly higher than those measured in the inflamed and healthy muscles (0.31 − 0.42% ID/cc). PET imaging further showed that D-[^18^F]FAla and D-[^18^F]FAla-*d*_*3*_ were able to differentiate bacterial infection from sterile inflammation. Both radiotracers had a similar ability to differentiate infection from inflammation with 2-fold signal difference. On the other hand, the two radiotracers showed similar increased bone uptake with time (∼1% ID/cc at 60 min), suggesting that the deuteration did not decrease the rate of in vivo defluorination. Through this study, it was demonstrated that D-[^18^F]FAla and D-[^18^F]FAla-*d*_*3*_ are useful PET tracers for detecting and monitoring bacterial infections to guide the clinical management of infected patients.

### [^68^Ga]Ga-NODAGA-SNA006: advancing non-invasive PET imaging of CD8^+^ T cells in immunotherapy

By Min Yang

Immunotherapy has revolutionized cancer treatment by enhancing the anti-tumour activity of CD8^+^ T cells, which are pivotal in immune response. CD8 expression is a critical biomarker for predicting clinical outcomes and guiding treatment decisions. However, conventional methods such as immunohistochemistry are invasive and static, limiting their capacity for real-time monitoring. [^68^Ga]Ga-NODAGA-SNA006, a site-specific radiolabelled nanobody (Wang et al. 2024a, b) offers a ground-breaking solution for non-invasive PET imaging of CD8^+^ T cells. In a human dose-escalation study conducted at the *First Affiliated Hospital of Soochow University*, this tracer demonstrated convenient preparation, excellent safety and tolerability, rapid clearance (t_1/2_ <30 min), and real-time, accurate imaging. Importantly, its performance aligns with immunohistochemistry, as ^68^Ga-uptake in tumours (SUV _mean_) showed a strong linear correlation with CD8^+^ T-cell infiltration in biopsy samples (R²=0.757, *p* = 0.011). These features enable dynamic monitoring of immune responses and evaluation of immunotherapy efficacy. [^68^Ga]Ga-NODAGA-SNA006 represents a transformative advance in cancer immunotherapy, offering the potential to non-invasively track CD8^+^ T cells, facilitate real-time treatment adjustments, and enhance personalized medicine. Further validation through larger clinical trials will establish its role in routine practice, bridging the gap between dynamic imaging and immune assessment to optimize immunotherapy strategies and improve patient outcomes.

### [^89^Zr]Zr-girentuximab for PET–CT imaging of clear-cell renal cell carcinoma: a prospective, open-label, multicentre, phase 3 trial

By Lars Perk

This highlight commentary discusses the use of [^89^Zr]Zr-DFO-girentuximab for imaging of clear cell renal cell carcinoma (ccRCC), the most common and aggressive form of kidney cancer (Shuch et al. 2024). Girentuximab, also known as cG250, has been in development for years, with the first publication on ^89^Zr-labelled cG250 dating back to 2004 (Brouwers et al. 2004). Girentuximab targets carbonic anhydrase IX (CAIX), which is overexpressed in ccRCC, making it a potential valuable tool for precise imaging and diagnosis.

The ZIRCON trial was a prospective, open-label, multicentre, phase 3 study conducted across 36 research hospitals in nine countries. Patients with indeterminate renal masses (≤ 7 cm) suspicious for ccRCC and scheduled for nephrectomy received a single dose of [⁸⁹Zr]Zr-DFO-girentuximab (37 MBq ± 10%; 10 mg girentuximab) followed by PET–CT imaging at day 5 (± 2 days). The primary endpoints were the sensitivity and specificity of the imaging to detect ccRCC, with histopathological confirmation as the standard of truth. [⁸⁹Zr]Zr-DFO-girentuximab was administered to 300 patients of whom 284 were evaluable. The mean age was 61 years, with 71% male participants. The mean sensitivity was 85.5% and the mean specificity was 87.0%. No significant safety concerns were observed.

The study concludes that [⁸⁹Zr]Zr-DFO-girentuximab PET–CT is a highly accurate, non-invasive imaging modality for detecting and characterizing ccRCC. The good diagnostic performance suggest that this imaging technique could impact clinical practice by improving early diagnosis, patient stratification, and reducing unnecessary invasive procedures.

After over two decades of development, [⁸⁹Zr]Zr-DFO-girentuximab (TLX250-CDx) is on the verge of becoming available for regular clinical use (Telix press release), which, if approved by the FDA, will be the first ⁸⁹Zr-labelled monoclonal antibody with a marketing authorization.

### NIR-II scattering gold superclusters for intravascular optical coherence tomography molecular imaging

By Peter Caravan

Intravascular optical coherence tomography (IV-OCT) is a clinical imaging technique used to visualize the coronary arteries and provides real time information on atherosclerotic plaques, thrombus, and placed stents. However, there are currently no molecular imaging agents for IV-OCT. In the highlighted paper, gold superclusters were developed whose physical properties were tailored to maximize the scattering of light at the 1350 nm IV-OCT laser line rendering the superclusters detectable by IV-OCT (Calvert et al. 2024). The clusters were derivatized with a ligand to P-selectin, an established vascular inflammation marker, and demonstrated that IV-OCT with these clusters could detect and stage vascular inflammation in a rat model. This paper highlights the potential of molecular probes that provide contrast in OCT, a largely unexplored area, and which could have applications beyond cardiology.

### Inspiring the future: youth engagement and bridging radiopharmaceutical sciences with radiobiology

By Peter Laverman

**Fig. 3:**
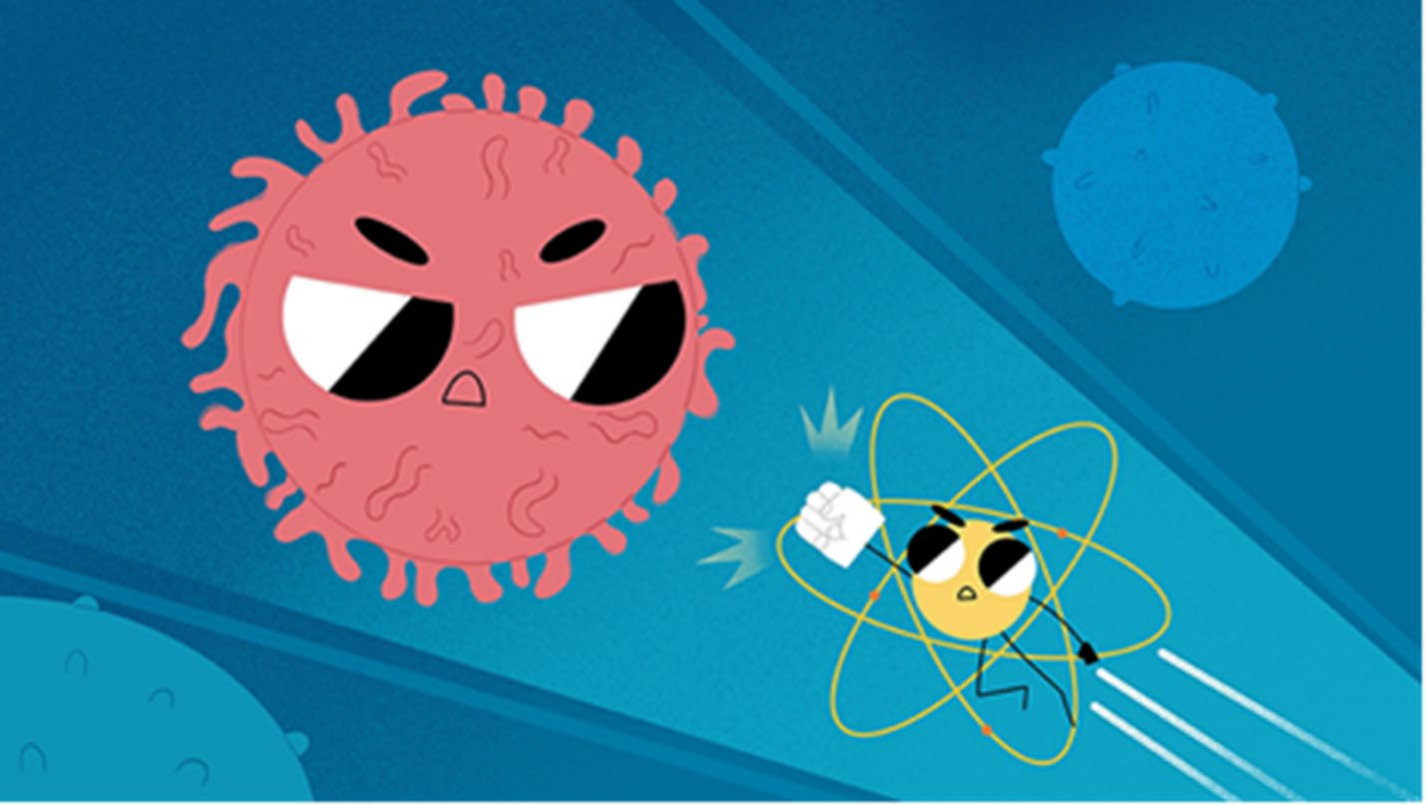
An exemplary image to explain attack of a tumour cell by a radiopharmaceutical for a young readership. Reproduced from Kleinendorst et al. under a Creative Commons Attribution License (CC BY) (Kleinendorst et al. 2024)

Next to the development of new and improvement of existing therapeutic radiotracers by radiochemists, knowledge on how these tracers specifically affect the tumour and its microenvironment is very important to improve therapy for patients. Radiobiology studies tailored to targeted radionuclide therapy plays an increasingly crucial role in this (Terry et al. 2019). However, besides addressing all these fundamental issues, even more important for the strongly growing field of nuclear medicine is to attract new and young professionals to ensure sufficient future interest and workforce (Scott et al. 2024). To gain the scientific interest of this next generation it is crucial to explain the developments in our field in an understandable, yet scientific way. Recently, a good example was published in Frontiers for Young Minds (Kleinendorst et al. 2024). This peer-reviewed scientific paper, also reviewed by two young reviewers (aged 10 and 13 years), explains targeted radionuclide therapy for an audience of 12–15 years old (Fig. 3). From explaining radioactivity and the difference between alpha, beta, and Auger electron emitters to the concept of targeting is explained and illustrated by PSMA-targeted radionuclide therapy in an easy to understand, but still scientific way. In conclusion, the field of radiopharmaceutical sciences should interact more with the radiobiological sciences, and radiopharmaceutical scientists should be aware that the colleagues of the future will only be found by presenting our work for a young audience in dedicated journals.

### Synthetic PET from CT improves diagnosis and prognosis for lung cancer

By Zhen Cheng

The highlighted article reports an innovative application of artificial intelligence (AI) to bridge the accessibility gap troubling the popularization of PET imaging (Salehjahromi et al. 2024). By leveraging a conditional generative adversarial network (cGAN), synthetic [^18^F]fluorodeoxyglucose PET (FDG-PET) images could be produced from corresponding CT scans, validated through a robust dataset of 1,478 multi-modal lung cancer imaging cases. The findings are impressive, with radiologists confirming the comparable quality and tumour contrast of synthetic PET images to real PET scans. Moreover, radiogenomics analyses reveal that synthetic PET imaging accurately capture dysregulated cancer hallmark pathways, further emphasizing its biological relevance.

The study highlights the potential clinical roles of synthetic PET in enhancing lung cancer diagnostics, staging, risk prediction, and prognosis - key areas where traditional PET excels but is often limited by cost and accessibility in resource-constrained settings. This work not only demonstrates the feasibility of high-fidelity PET synthesis but also underscores the broader implications of integrating AI-driven solutions into practical medicine.

While promising, further investigation into real-world implementation, scalability, and generalizability across different cancer types is necessary. Overall, this study provides a compelling proof-of-concept for AI’s role in making advanced medical imaging more accessible, potentially improving cancer diagnosis workflows in resource-limited scenarios, such as vast underdeveloped countries.

### Cyclotron production of manganese-52 for PET/MRI imaging, and update of the SPES project for medical isotope production

By Cornelia Hoehr.

The integration of Positron Emission Tomography (PET) with Magnetic Resonance Imaging (MRI) offers enhanced diagnostic capabilities. Manganese-52 emerges as a promising dual-purpose agent due to its paramagnetic and radioactive properties, being the topic of the research project METRICS (*Multimodal pET/mRI Imaging with Cyclotron-produced*^*51/52*^*Mn (β*^+^*emitter/paramagnetic) isotopes*).

To utilize the ⁵²Cr(p, n)⁵²Mn reaction on a medical cyclotron, natural and enriched Cr targets were produced using Spark Plasma Sintering (SPS) (Porto et al. 2024). ^52^Mn was isolated with a recovery rate of 75–78% after 4 h irradiation in a medical cyclotron, with a beam energy of 16.8 MeV and beam currents between 10 and 50 microA. The purified ^52^Mn was complexed with chelators, including DOTA-SCN and DASD ligands, achieving radiochemical purities exceeding 95%. Phantom imaging was successfully conducted using microPET and clinical MRI systems.

The METRICS project is part of LARAMED (LAboratory of RAdionuclides for MEDicine). LARAMED is also pursuing medical isotope production at the SPES (Selective Production of Exotic Species) project at the INFN-Legnaro National Laboratories (LNL), Italy, investigating novel radioisotopes like ^67^Cu, ^47^Sc, and ^149/152/155/161^Tb for theranostic applications.

**Fig. 4:**
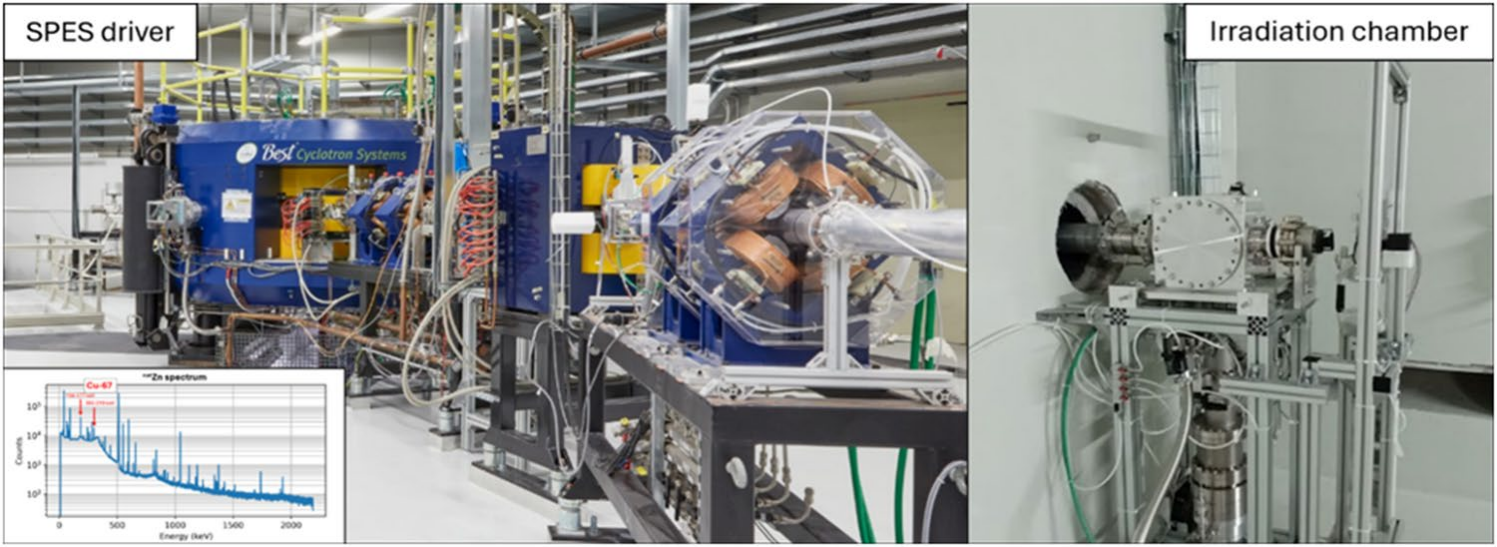
The SPES cyclotron, irradiation chamber and gamma spectroscopy spectrum of the production of ^67^Cu (image courtesy of F. Azaiez, personal communication)

In recent years, the SPES cyclotron underwent significant upgrades, including improvements to its ion source and RF systems. Following its recommissioning, the cyclotron successfully performed initial experiments using proton beams with energies of 35, 50, and 70 MeV. Notably, in November 2024, SPES achieved a major milestone by producing and transporting its first low-energy radioactive beam, completing Phase 1 of its development, see Fig. 4.

### ^52^Mn-labelled trastuzumab as new PET probe for HER2 overexpressing tumour

By Tamer Sakr

^52^Mn-labelled trastuzumab, as a new PET radiopharmaceutical for human epidermal growth factor receptor 2 (HER2) tumour receptor, has been developed and evaluated (Omweri et al. 2024). Trastuzumab plays a crucial role for the treatment of HER2 overexpressing tumours, acting as a monoclonal therapeutic agent with high affinity to HER2 receptor (Ducharme et al. 2023). Conjugating manganese-52 with trastuzumab allows accurate and long duration visualization of HER2 overexpressing tumours, offering better insights into tumour progression and treatment efficacy over time. Conjugation of manganese-52 with trastuzumab, to give a PET tracer suitable for longer time point imaging (Lau et al. 2020), was optimized and evaluated to ensure efficacy and stability (Omweri et al. 2024). ^52^Mn-labelled trastuzumab showed high in vivo stability and efficient targeting ability for HER2 overexpressing tumours in animal models, mice bearing BT474 tumour, acting as efficient PET radiotracer capable of tracking tumour growth and evaluating therapeutic outcomes with time. ^52^Mn-labelled trastuzumab was presented as a potential imaging agent for HER2 overexpressing tumours with the potential to offer better diagnosis, treatment planning and monitoring treatment response.

### Palladium-103 (^103^Pd/^103m^Rh), a promising Auger electron emitter for targeted radionuclide therapy of disseminated tumour cells– absorbed doses in single cells and clusters, with comparison to ^177^Lu and ^161^Tb

By Jan Rijn Zeevaart

It is postulated that in order to offer cancer patients a cure (not only palliative care), one needs to eradicate disseminated tumour cells. This can potentially be achieved via targeted radionuclide therapy (TRT) but it requires the appropriate radionuclides to provide high enough absorbed doses in single cells. ^161^Tb with its additional Auger electrons (commonly referred to as ^177^Lu plus (^177^Lu+) as the rest of the physical characterisations are virtually identically to ^177^Lu) has advantages over ^177^Lu which have been proven in vitro and preclinically while clinical trials are now ongoing to show if this translate to the clinic, for instance the PROGNOSTICS study (Chirindel et al. 2024).

The apparent success of ^161^Tb re-sparked interest in Auger Emitters (AE). One such AE-emitter radioisotope is palladium-103 (^103^Pd). When considering ^103^Pd for TRT, it is important to note that ^103^Pd decays (T_1/2_ = 16.991 d) by electron capture into rhodium-103m (^103m^Rh) which, in turns, decays (T_1/2_ = 56.12 min) through isomeric transition into stable ^103^Rh. The combination as ^103^Pd/^103m^Rh is an in vivo generator and one assumes that both deliver their radiation dose to cells.

The Monte Carlo track structure code CELLDOSE was used to assess absorbed doses in single cells and clusters of 19 cells (Hindié et al. 2024). Through modelling the radionuclide was distributed on the cell surface, within the cytoplasm, and in the nucleus. Absorbed doses from ^103^Pd/^103m^Rh, ^177^Lu and ^161^Tb were then compared.

**Fig. 5:**
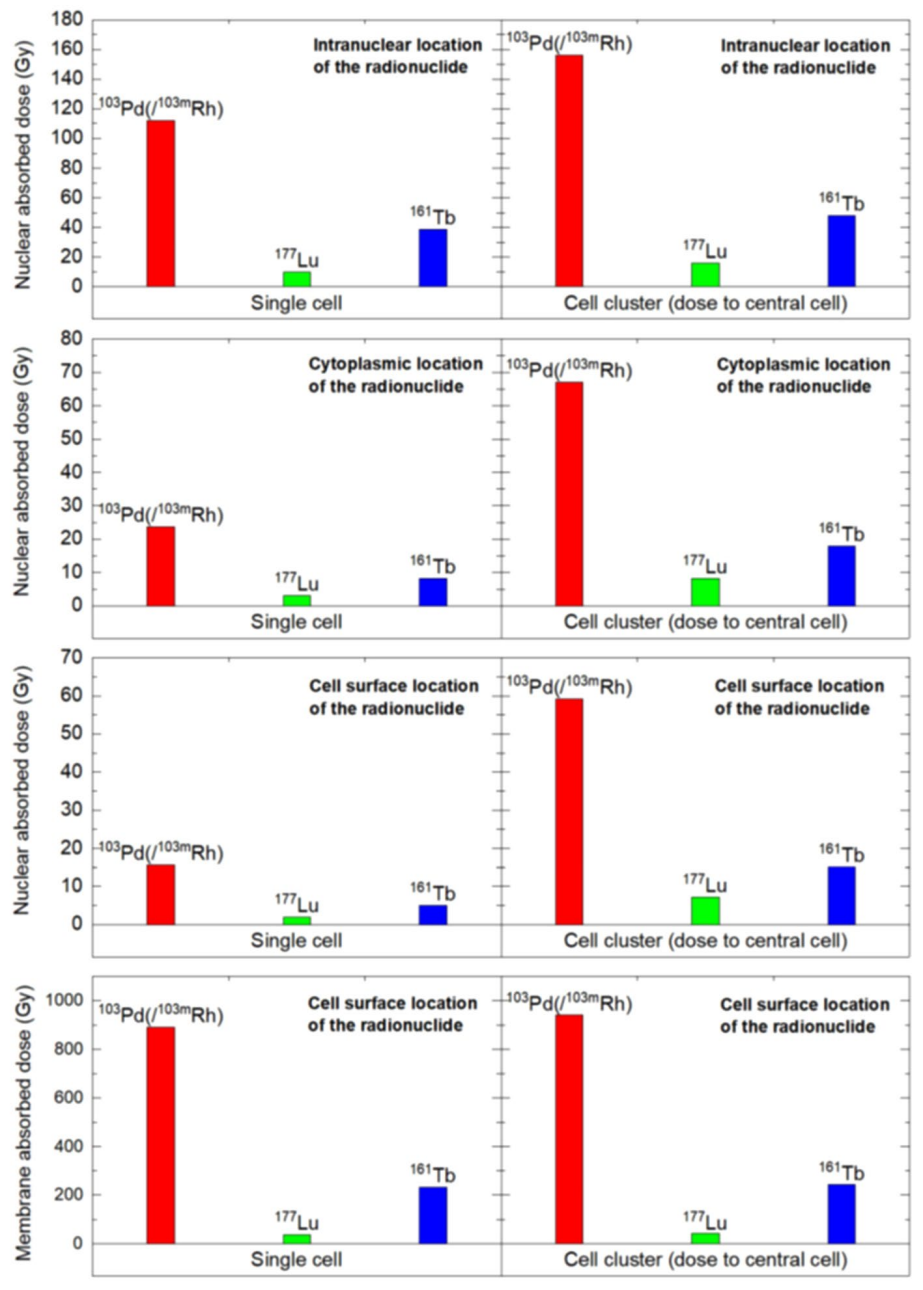
Nuclear and membrane absorbed doses to the single cell and central cell of a 19 cells-cluster, considering various distributions of 103Pd(/103mRh) (red), 177Lu (green), and 161 Tb (blue). Reprinted from Chirindel et al. under a Creative Commons Attribution 4.0 International License (Chirindel et al. 2024)

The authors found that in the 19-cell cluster, nuclear absorbed doses were 7.1 to 9.9-fold higher with ^103^Pd/^103m^Rh than with ^177^Lu, while ^161^Tb yielded intermediate values. The results for the single cell and cell cluster within the various cellular compartment are summarized in Fig. 5. Importantly the authors took as reference point the data regarding the CA20948 cell line exposed to ^177^Lu-DOTATATE, which indicated that the survival fraction is below 0.01 when the dose to cell nuclei is above 7.3 Gy (O’Neill et al., 2020). Furthermore, it has recently been established that not only the cell nucleus is a target for AE emitters but also the cell membrane (Borgna et al. 2022). With ^103^Pd/^103m^Rh located on the cell surface, the cell membrane dose was ~ 4 times higher than with ^161^Tb, and ~ 25 times higher than with ^177^Lu, with 96% contribution from AE. As depicted in Fig. 5, the absorbed dose is higher for ^103^Pd/^103m^Rh in every compartment and this is more pronounced for the cell clusters. For the cell membrane absorbed dose (row 3), as opposed to the nuclear absorbed dose (row 4), ^103^Pd/^103m^Rh over ^177^Lu and ^161^Tb, is the highest.

In conclusion ^103^Pd, a next-generation Auger emitter, can deliver substantially higher absorbed doses than ^177^Lu (and, to a lesser extent, than ^161^Tb) to single tumour cells and cell clusters.

## Data Availability

Datasets mentioned in this article can be found in the cited articles.

## Abbreviations

AE: Auger emitter
AI: Artificial Intelligence
ccRCC: Clear cell renal cell carcinoma
DNA: Deoxyribonucleic acid
FAPI: Fibroblast activating protein inhibitor
HER: Human epidermal growth factor
IDP: Intrinsically disordered proteins
IV-OCT: Intravascular optical coherence tomography
mCRPC: Metastatic castrate-resistant prostate cancer
MRI: Magnetic resonance imaging
OCT: Optical coherence tomography
PET: Positron emission tomography
PSMA: Prostate specific membrane antigen
RESCA: Restrained Complexing Agent
SiFA: Silicon-Based Fluoride Acceptor
SPECT: Single photon emission computed tomography
SUV: Standard uptake value
TRT: Targeted radionuclide therapy
