# Correction: Highlight selection of radiochemistry and radiopharmacy developments by editorial board

**DOI:** 10.1186/s41181-025-00351-w

**Published:** 2025-06-02

**Authors:** S. Spreckelmeyer, J. Dasilva, C. Decristoforo, R. H. Mach, J. Passchier, G. Carlucci, M. Al Qahtani, A. Duatti, B. T. Cornelissen, J. Engle, A. Denkova, J. J. M. A. Hendrikx, Y. Seimbille, X. Yang, H. Jia, M-R. Zhang, M. Yang, L. Perk, P. Caravan, P. Laverman, Z. Cheng, C. Hoehr, T. Sakr, J. R. Zeevaart

**Affiliations:** 1 Department of Nuclear Medicine, Charité - Universitätsmedizin Berlin, Corporate Member of Freie Universität Berlin, Humboldt- Universität zu Berlin, Berlin Institute of Health, Augustenburger Platz 1, 13353 Berlin, Germany; 2 University of Montreal, Montreal, Canada; 3 Medical University Innsbruck, Innsbruck, Austria; 4 University of Pennsylvania, Philadelphia, USA; 5 Invicro London, London, UK; 6 University of California, Los Angeles, CA USA; 7 Cyclotron and Radiopharmaceuticals Department, Research & Innovation, King Faisal Specialist Hospital and Research Center, Riyadh, Saudi Arabia; 8 University of Ferrara, Ferrara, Italy; 9 Oxford University, Oxford, UK; 10 University Medical Center Groningen, Groningen, Netherlands; 11 University of Wisconsin, Madison, WI USA; 12 Delft University of Technology, Delft, Netherlands; 13 NKI, Amsterdam, The Netherlands; 14 Erasmus MC, Rotterdam, Netherlands; 15 Peking University First Hospital, Beijing, China; 16 Beijing Normal University, Beijing, China; 17 National Institutes for Quantum Science and Technology, Chiba, Japan; 18 Jiangsu Institute of Nuclear Medicine, Jiangsu, China; 19 Radboud University Medical Center Nijmegen, Nijmegen, Netherlands; 20 Massuchusetts General Hospital, Harvard University, Boston, USA; 21 Radboud Medical Center, Nijmegen, Netherlands; 22 Shanghai Institute of Materia Medica, Chinese Academy of Sciences, Shanghai, China; 23 TRIUMF, Vancouver, BC Canada; 24 Egyptian Atomic Energy Authority, Cairo, Egypt; 25 Necsa, Pretoria, South Africa

**Correction: EJNMMI Radiopharmacy and Chemistry (2025) 10:13**

10.1186/s41181-025-00335-w

The original publication of this article contained an incorrect author name. The incorrect and correct information is listed in this correction article. The original article has been updated.

Incorrect

M. Al Qhatani

Correct

M. Al Qahtani 

